# Delivering the second revolution in site-specific nucleases

**DOI:** 10.7554/eLife.02904

**Published:** 2014-05-08

**Authors:** Perry B Hackett, Nikunj V Somia

**Affiliations:** 1 **Perry B Hackett** is in the Department of Genetics, Cell Biology and Development, University of Minnesota, Minneapolis, United Stateshacke004@umn.edu; 2 **Nikunj V Somia** is in the Department of Genetics, Cell Biology and Development, University of Minnesota, Minneapolis, United States

**Keywords:** protein transduction, zinc-finger nucleases, transcription activator-like effector nucleases, lentiviral vector, gag, gene therapy, human, viruses

## Abstract

Viruses have been used to deliver two types of site-specific nucleases into cells for targeted gene editing.

**Related research article** Cai Y, Bak RO, Mikkelsen JG. 2014. Targeted genome editing by lentiviral protein transduction of zinc-finger and TAL-effector nucleases. *eLife* **3**:e01911. doi: 10.7554/eLife.01911

**Image** A schematic of a virus containing site-specific nucleases (blue and green dots) and templates (red squiggles) for gene editing

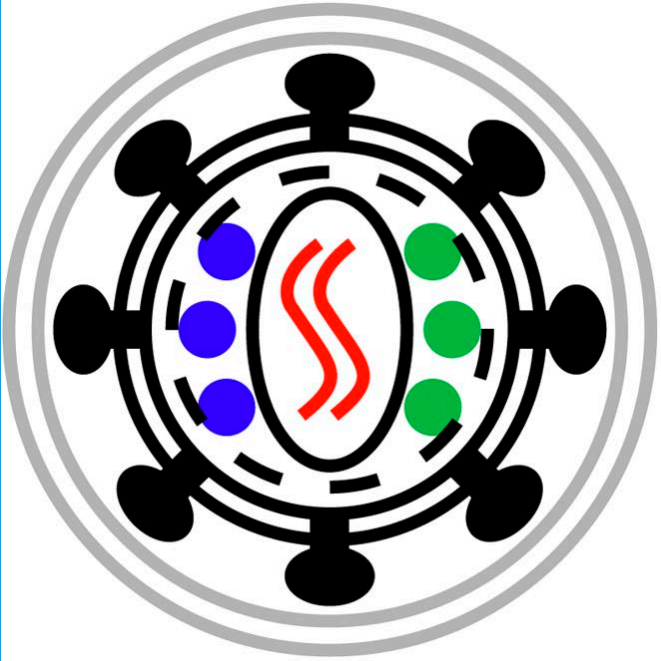

Microorganisms rely on enzymes called restriction endonucleases to protect them against viruses. These restriction enzymes work by breaking the DNA in the virus into short fragments that are no longer active (Linn and Arber, 1968). When isolated and purified, these restriction enzymes can also be used to cut DNA from different sources into fragments that can then be recombined into new sequences with specific properties. When site-specific endonucleases were first used to fashion recombinant DNA molecules in the early 1970s, they revolutionised research in molecular and cellular biology (Cohen et al., 1973; Cohen, 2013). The power of this approach was immediately recognised, but worries about potential biohazards (Berg et al., 1974) suppressed the technology for a couple of years, before common sense and appropriate experimentation demonstrated its safety (Berg and Singer, 1995).

The use of naturally occurring restriction endonucleases limited what could be achieved by gene therapy. These enzymes generally cleave DNA molecules after every few hundred to few thousand base pairs, which is good enough for constructing recombinant DNAs for use in research. Gene therapy, on the other hand, requires the ability to cleave a DNA molecule at a specific site in a genome comprising billions of basepairs.

Forty years after restriction enzymes were first used to cleave DNA strands, a new revolution employing artificial site-specific nucleases has overtaken biological sciences. This began with the creation of hybrid enzymes, composed of protein sequences called zinc fingers and an endonuclease domain taken from a naturally occurring restriction enzyme (Kim et al., 1996). Like a natural restriction enzyme, a zinc finger nuclease (ZFN) operates as a dimer, with one zinc finger binding to each strand of a double-stranded DNA molecule. With ZFNs cleaving both strands of DNA at the same place, researchers can either disable a gene, or introduce specific changes in the sequence by adding a ‘template DNA’ molecule (Bibikova et al., 2002) as part of a process called homology-directed repair (Urnov et al., 2010). These processes are called gene editing.

Since the introduction of ZFNs, two more highly adaptable methods have been developed. The first method employs TALENs (transcription activator-like endonucleases), which are like ZFNs but use alternative pairs of polypeptides in place of the zinc fingers (Bogdanove and Voytas, 2011). The second method, called CRISPR-Cas, cleaves specific DNA sequences that have been targeted by designer RNA sequences (Haurwitz et al., 2010). TALENs and the CRISPR-Cas system are considered easier to engineer and more reliable than ZFNs (Gaj et al., 2013). Now, in *eLife*, Yujia Cai, Rasmus Bak and Jacob Mikkelsen of Aarhus University in Denmark present a new method that could bring these gene-editing techniques a step closer to mainstream medical use (Cai et al., 2014).

Despite the immense potential of gene editing, deploying it has been problematic. For many types of gene therapy, proteins and the nucleic acid molecules that encode ZFN, TALEN and CRISPR activities must be introduced into the cells of an individual. However, the immune response protects all the cells in the body from invasion, so until the immune system can be effectively controlled to prevent this response, most gene editing is limited to single cells. This has certain advantages: several molecular transfer technologies can be used to introduce DNA molecules, RNA molecules or the proteins that direct DNA cleavage into cells. Moreover, working with single cells also offers the opportunity to screen gene-edited genomes for unwanted mutations that can arise when the nucleases cut the DNA at a different, related sequence to the one that should be targeted. If just the right amounts of nuclease and template are introduced into a cell, this ‘off-targeting’ can be reduced—which is important for ensuring gene therapy is safe.

Viruses called integration- and replication-deficient lentiviruses (IDLVs) can penetrate the cellular barriers to introduce precise levels of site-specific nucleases and templates into cells (Cornu and Cathomen, 2007; Lombardo et al., 2007). These viruses are highly engineered versions of human immunodeficiency virus type 1 (HIV-1). Appropriately modified IDLVs are able to infect a range of cell types and deliver both their genetic cargos and their viral proteins. Thus, genes encoding ZFNs and templates for homology directed repair could be delivered to cells in whole animals (Joglekar et al., 2013).

Cai, Bak and Mikkelsen hypothesised that they could deliver functional nucleases in the lentiviral particles while blocking the integration of viral DNA into the genome (Cai et al., 2014). The technique exploits the fact that viruses are made from long proteins that are split into shorter, functional proteins by a viral enzyme. In this case, Cai et al. fused either ZFN- or TALEN-nuclease encoding sequences to the *gag* (group-specific antigens) set of genes in the IDLVs (Figure 1). This combined genetic sequence created a single ‘fusion protein’, which could cut out specific target genes in several mammalian cell lines. In addition, using fusion proteins makes it possible to control the level of nuclease polypeptides entering cells simply by introducing different numbers of viral particles. This contrasts with previously used methods that instead deliver the genes encoding ZFNs or TALENs. As these genes are expressed at variable levels inside the cell, depending on the stability and integration of the delivered DNA, precise control of the number of nucleases is not possible.

**Figure 1.**
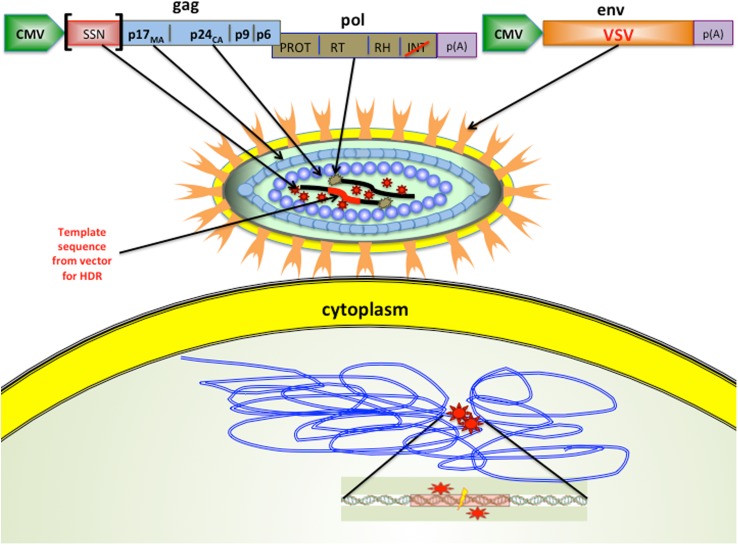
Modified viruses can deliver the enzymes and templates needed for gene editing. The lentiviral particles created by Cai et al. had glycoproteins (VSV; pale orange) on their surface, which enabled them to infect of a wide range of cell types. The lentiviral particles also contained site-specific nucleases (thick black lines) that targeted specific sequences in the DNA of the host cell, and a DNA template (thick red line) that was added to the genome of the host cell. Cai et al. demonstrated that their approach worked with two types of site-specific nuclease (SSN): zinc finger nucleases (ZFNs) and transcription activator-like endonucleases (TALENs). The modified lentiviral ‘genome’ is illustrated at the top: when this is expressed as a polypeptide inside the host cell, it is cleaved by protease (PROT; red stars). The gene for the SSN was inserted into the *gag* sequence of genes in the virus. Cai et al. also made a number of other important modifications: the normal lentiviral promoter that marks the start of a gene was replaced by a different promoter (called CMV), and the gene in the *pol* sequence that encodes an enzyme called integrase (INT) was modified (red slash) to inactivate the integration activity. The virus used was actually an RNA virus, so reverse transcriptase (RT; brown stars) was used to convert the RNA into DNA. The bottom of the figure shows the DNA of the host cell. The SSNs bind to the DNA (double blue lines) at the sites indicated by the red stars, and the cleavage site is shown by the lightning bolt. VSV: vesicular stomatitis virus; CMV: cytomegalovirus.

Mikkelsen and colleagues demonstrated that genes in up to about 20% of cultured cells could be disrupted by IDLV-mediated protein transfer, and that the incoming viral genomes could provide DNA templates to support specific gene editing, albeit at a lower rate than cleavage of the DNA target site. However, the rates of TALEN-mediated gene editing were substantially reduced compared with that by ZFNs (due to the polypeptides in the TALEN being inactivated).

Although the rates of gene editing reported by Cai et al. are low and variable, they approach the levels required for effective gene therapy in whole animals—including humans. As this is the first demonstration of the use of gag-modified IDLVs for gene editing in animal cells, there is room for improving the design of the IDLVs and the way they are delivered. One challenge is to eliminate the possibility of a recombination event occurring between a circular form of the template, which would lead to unwanted sequences being inserted into the chromosome. The platform could also be adapted to work with the CRISPR system. Moreover, Mikkelsen and colleagues speculate on the basis of preliminary data that this method of delivery of site-specific nucleases may reduce off-targeting rates through its ability to carefully control the levels of nuclease in the cells.

The aim of researchers during the original site-specific endonuclease revolution, to repair parts of the human genome using gene editing techniques, was derided some years ago as ‘not only naïve but also wrong-headed’ (Lewin, 1970). This second revolution brings us closer to proving that notion wrong.
